# A promoter variant of the *APOA5* gene increases atherogenic LDL levels and arterial stiffness in hypertriglyceridemic patients

**DOI:** 10.1371/journal.pone.0186693

**Published:** 2017-12-06

**Authors:** Minjoo Kim, Minkyung Kim, Hye Jin Yoo, Eunji Lee, Jey Sook Chae, Sang-Hyun Lee, Jong Ho Lee

**Affiliations:** 1 Research Center for Silver Science, Institute of Symbiotic Life-TECH, Yonsei University, Seoul, Korea; 2 Department of Food and Nutrition, Brain Korea 21 PLUS Project, College of Human Ecology, Yonsei University, Seoul, Korea; 3 National Leading Research Laboratory of Clinical Nutrigenetics/Nutrigenomics, Department of Food and Nutrition, College of Human Ecology, Yonsei University, Seoul, Korea; 4 Department of Family Practice, National Health Insurance Corporation, Ilsan Hospital, Goyang, Korea; University of Milano, ITALY

## Abstract

Hypertriglyceridemia is recognized as an independent risk factor for coronary artery disease. The apolipoprotein A5 gene (*APOA5*) is a key regulator of triglyceride levels. We aimed to evaluate the associations of single nucleotide polymorphisms (SNPs) in *APOA5*, including -1131T>C and c.553G>T, with hypertriglyceridemia, apoA5 concentrations, atherogenic LDL cholesterol levels, and arterial stiffness in hypertriglyceridemic patients. The study population included 599 hypertriglyceridemic patients (case) and 1,549 untreated normotriglyceridemic subjects (control). We genotyped two *APOA5* variants, -1131T>C (rs662799) and c.553G>T (rs2075291). The frequencies of the CC genotype of -1131T>C (0.165) and the T allele of c.553G>T (0.119) were significantly higher in hypertriglyceridemic patients than in normotriglyceridemic subjects (0.061 and 0.070, respectively; all *p*<0.001). In the control and case groups, both the -1131T>C and c.553G>T variants were associated with higher triglyceride and lower HDL cholesterol levels. Controls with the -1131CC variant had lower apoA5 concentrations than controls with the -1131TT variant. Similar effects of the -1131T>C variant on apoA5 were observed in the cases. In the hypertriglyceridemic group, the -1131T>C variant was associated with a smaller LDL particle size, higher levels of oxidized LDL and malondialdehyde, and higher brachial-ankle pulse wave velocity. The -1131T>C and c.553G>T polymorphisms were associated with hypertriglyceridemia in the study population, but only the -1131T>C polymorphism directly affected apoA5 concentrations. Hypertriglyceridemic patients carrying the *APOA5* -1131T>C polymorphism exhibited increased atherogenic LDL levels and arterial stiffness, probably due to an effect of the -1131T>C polymorphism on apoA5 concentrations.

## Introduction

Hypertriglyceridemia is recognized as an independent risk factor for coronary artery disease (CAD) [1,2]. Consistent with this notion, the 1131T>C single nucleotide polymorphism (SNP) in the apolipoprotein A5 gene (*APOA5*) is associated with an increased risk of CAD in several ethnic populations, probably due to its association with hypertriglyceridemia [3–5]. However, limited data are available regarding the association between the *APOA5* locus and a phenotypic manifestation of atherosclerosis in humans. Brachial-ankle pulse wave velocity (ba-PWV) is correlated with aortic PWV [6], is a predictor for cardiovascular morbidity and mortality [7], and has been suggested to be a composite risk factor for the identification of subjects with early atherosclerotic changes [6].

Both apolipoprotein A5 (apoA5) and *APOA5* are key regulators of plasma triglyceride (TG) levels [8–11]. In many human populations, *APOA5* SNPs are associated with variations in serum TG levels. Among the five commonly studied *APOA5* SNPs (-1131T>C, -3A>G, 56C>G, IVS3+476G>A, and 1129T >C) [12–14], -1131T>C and 56C>G are considered functional tag SNPs [15–17]; however, the minor 56G allele is not detected in the Korean population [18]. The c.553G>T (p.185Gly>Cys, rs2075291) SNP is associated with marked hypertriglyceridemia among Asians in some studies; specifically, T allele carriers in Asian populations have a 4-fold higher risk of high TG levels [19–21]. Therefore, we aimed to evaluate the association of *APOA5* SNPs, including -1131T>C and c.553G>T, with hypertriglyceridemia and apoA5 concentrations in a Korean population. We also investigated the variations at the *APOA5* gene locus encoding apoA5, which correlates with a more atherogenic pattern of low-density lipoprotein (LDL) cholesterol levels and arterial stiffness in hypertriglyceridemic patients.

## Materials and methods

### Study population

Two thousand one hundred sixty-seven study participants with nondiabetic normotriglyceridemia (TG levels<150 mg/dL) and hypertriglyceridemia (TG levels≥150 mg/dL) were recruited for this study from the Health Service Center (HSC) during routine checkups at the National Health Insurance Corporation Ilsan Hospital in Goyang, Korea (January 2010-March 2015). Based on data from the HSC, subjects who agreed to participate in the study were referred to the Department of Family Medicine or Internal Medicine, and their health and serum lipid profiles were rechecked. The exclusion criteria were a current diagnosis or history of cardiovascular disease, liver disease, renal disease, pancreatitis, or cancer; and regular use of any medication. The aim of the study was carefully explained to all participants, who then provided written informed consent. The Institutional Review Board of Yonsei University and the National Health Insurance Corporation Ilsan Hospital approved the study protocol, which complied with the Declaration of Helsinki.

### Laboratory experiments

General anthropometry, blood pressure (BP) measurements, and sample collection procedures are described elsewhere [22]. Participants’ body weights and heights were measured, and their body mass indexes (BMIs) were calculated in units of kilograms per square meter. BP was measured twice with an automatic BP monitor (FT-200S; Jawon Medical, Gyeongsan, Korea) after a resting period of at least 20 min, and the average value was used. Blood samples were collected following an overnight fast of at least 12 hours.

The levels of fasting TGs, total cholesterol, high-density lipoprotein (HDL) cholesterol, LDL cholesterol, and glucose were analyzed using an autoanalyzer (Hitachi 7600 Autoanalyzer, Hitachi Ltd., Tokyo, Japan). LDL particle size and oxidized (ox)-LDL and malondialdehyde (MDA) levels were measured using previously described methods [22]. Plasma apoA5 concentrations were measured using an enzyme immunoassay (Human apoA5 ELISA Kit; Cusabio, Zhejiang, China). The absorbance of the resulting color was measured at 450 nm using a VersaMax ELISA microplate reader (Molecular Devices, Sunnyvale, CA, USA). The ba-PWV was measured with an automatic waveform analyzer (model VP-1000; Nippon Colin Ltd., Komaki, Japan).

### Affymetrix Axiom™ KORV1.0–96 array hybridization and SNP selection

Two thousand one hundred sixty-seven samples were genotyped using the Axiom^®^ 2.0 Reagent Kit (Affymetrix Axiom^®^ 2.0 Assay User Guide; Affymetrix, Santa Clara, CA, USA) according to the manufacturer’s protocol. Approximately 200 ng of genomic DNA (gDNA) was amplified and randomly fragmented into 25- to 125-base pair (bp) fragments. The gDNA was initially amplified in a 40-μL reaction containing 20 μL of a 10 ng/μL genomic DNA stock and 20 μL of a master mix. The initial amplification reaction conditions were a 10-min initial amplification at room temperature, followed by amplification with 130 μL of Axiom 2.0 Neutral Solution, 225 μL of Axiom 2.0 Amp Solution and 5 μL of Axiom 2.0 Amp Enzyme. The amplification reactions were performed for 23±1 hours at 37°C. The amplification products were analyzed in an optimized reaction to amplify fragments between 200 and 1100 bp in length. A fragmentation step reduced the amplified products into segments of approximately 25–50 bp in length, which were end-labeled using biotinylated nucleotides. Following hybridization, the bound target was washed under stringent conditions to remove non-specific background and to minimize the background noise caused by random ligation events. Each polymorphic nucleotide was queried in a multicolor ligation event conducted on the array surface. After ligation, the arrays were stained and imaged on a GeneTitan MC Instrument (Affymetrix, Santa Clara, CA, USA). The images were analyzed using the Genotyping Console™ Software (Affymetrix, Santa Clara, CA, USA). The genotype data were produced using the Korean Chip (K-CHIP) available through the K-CHIP consortium. The K-CHIP was designed by the Center for Genome Science at the Korea National Institute of Health (4845–301, 3000–3031).

Samples with the following characteristics were excluded: sex inconsistency, markers with a high missing rate (>5%), individuals with a high missing rate (>10%), minor allele frequency <0.01, and a significant deviation from Hardy-Weinberg equilibrium (HWE) (*p*<0.001). SNPs in linkage disequilibrium with each other were excluded; therefore, the remaining 394,379 SNPs and 2,159 samples were used in subsequent association analyses.

### Statistical analysis

Descriptive statistical analyses were performed using SPSS version 23.0 (IBM, Chicago, IL, USA). The skewed variables were transformed to logarithmic form, and a two-tailed *p*-value <0.05 was considered statistically significant. An independent *t*-test was performed on the continuous variables to compare parameters between the controls and cases. HWE was assessed using PLINK version 1.07 (http://pngu.mgh.harvard.edu/purcell/plink/). The association between SNPs and TG levels was evaluated using a linear regression analysis. The frequency was tested using the chi-square test. The association of hypertriglyceridemia with a genotype was determined using the odds ratio (OR) [95% confidence intervals (CIs)] of a logistic regression model after adjusting for confounding factors. One-way ANOVA followed by Bonferroni *post hoc* test was performed to compare the differences among the *APOA5* -1131T>C genotypes in the normotriglyceridemic controls and hypertriglyceridemic patients.

## Results

A total of 394,379 SNPs and 2,159 samples were used in the analyses. The associations between genotypes and TG levels were evaluated with a linear regression analysis after adjusting for age and sex. Among the ten SNPs with the strongest association with TG levels, *APOA5* -1131T>C and *APOA5* c.553G>T exhibited the top two strongest associations (*p* = 4.83E-27 and *p* = 8.75E-13, respectively). In addition, the coverage of *APOA5* -1131T>C and *APOA5* c.553G>T was 99.7% and 99.8%, respectively. Therefore, we performed an association analysis using these two SNPs. Among 2,159 subjects, both *APOA5* variants were genotyped in 2,148 subjects; therefore, 11 subjects were excluded from this study.

The clinical and biochemical characteristics of normotriglyceridemic controls (*n* = 1,549) and hypertriglyceridemic cases (*n* = 599) are shown in Table 1. Case subjects were significantly older, more often male, heavier, and more often smokers than the controls. After adjusting for age, sex, BMI, smoking, and drinking, the hypertriglyceridemic subjects exhibited higher systolic and diastolic BPs; higher levels of TG, total cholesterol and ox-LDL; lower levels of HDL cholesterol, LDL cholesterol and apoA5; and smaller LDL particle sizes (Table 1).

**Table 1:** Clinical and biochemical characteristics of controls and hypertriglyceridemic patients.

	Controls (*n* = 1,549)		Cases (*n* = 599)		*p*^*a*^	*p*^*b*^
Age (year)	49.1	±0.29	50.8	±0.45	0.002	-
Male/Female, n (%)	564 (36.4)/985 (63.6)		300 (50.1)/299 (49.9)		<0.001	-
BMI (kg/m^2^)	23.7	±0.08	25.3	±0.12	<0.001	-
Current smoker, n (%)	204 (13.2)		121 (20.2)		<0.001	-
Current drinker, n (%)	920 (59.5)		372 (62.3)		0.241	-
Systolic BP (mmHg)	120.4	±0.0	126.0	±0.63	<0.001	0.001
Diastolic BP (mmHg)	75.0	±0.28	80.1	±0.44	<0.001	<0.001
Triglyceride (mg/dL)^*∮*^	89.5	±0.79	223.4	±3.34	<0.001	<0.001
Total cholesterol (mg/dL)^*∮*^	193.9	±0.89	208.9	±1.50	<0.001	<0.001
HDL cholesterol (mg/dL)^*∮*^	55.6	±0.34	46.2	±0.45	<0.001	<0.001
LDL cholesterol (mg/dL)^*∮*^	120.6	±0.81	120.2	±1.46	0.413	0.022
Glucose (mg/dL)^*∮*^	96.6	±0.55	100.6	±0.96	<0.001	0.346
Apolipoprotein A5 (ng/mL)^*∮*^	287.0	±6.94	229.2	±8.87	<0.001	<0.001
LDL particle size (nm)^*∮*^	24.1	±0.02	23.4	±0.08	<0.001	<0.001
Oxidized LDL (U/L)^*∮*^	45.6	±0.55	48.9	±0.84	<0.001	0.003
Malondialdehyde (nmol/mL)^*∮*^	8.97	±0.11	9.57	±0.17	<0.001	0.081
ba-PWV (cm/s)^*∮*^	1,300.7	±7.71	1,371.3	±16.9	<0.001	0.149
***APOA5* -1131T>C, *n* (%)**						
TT	807 (52.1)		214 (35.7)		<0.001	
TC	648 (41.8)		286 (47.7)			
CC	94 (6.1)		99 (16.5)			
C allele frequency	836 (27.0)		484 (40.4)		<0.001	
***APOA5* c.553 G>T, *n* (%)**						
GG	1,335 (86.2)		464 (77.5)		<0.001	
GT	210 (13.6)		128 (21.4)			
TT	4 (0.3)		7 (1.2)			
T allele frequency	218 (7.0)		142 (11.9)		<0.001	

Means ± standard errors (SE).

^*∮*^Tested using logarithmic transformation.

*p*-values were derived from an independent *t*-test.

*p*^*a*^, Unadjusted;

*p*^*b*^, Adjusted for age, sex, BMI, smoking, and drinking.

### Distribution of *APOA5* -1131T>C and *APOA5* c.553G>T polymorphisms

Genotypic distributions of the *APOA5* -1131T>C and *APOA5* c.553G>T polymorphisms in the entire study population were in HWE. The genotypic distribution of the -1131T>C polymorphism was significantly different between normotriglyceridemic controls and hypertriglyceridemic patients (*p*<0.001). The minor C allele frequency was significantly increased in hypertriglyceridemic participants (0.404) compared with that in controls (0.270; *p*<0.001; Table 1). Similarly, the genotypic distribution of the c.553G>T polymorphism was significantly different between normotriglyceridemic controls and hypertriglyceridemic patients (*p*<0.001). The minor T allele frequency was significantly increased in hypertriglyceridemic participants (0.119) compared with that in controls (0.070; *p*<0.001; Table 1). Regarding the *APOA5* c.553G>T polymorphism, we pooled heterozygotes (GT) and rare allele homozygotes (TT) to increase the statistical power. Additionally, since the -1131T>C and c.553G>T polymorphisms were not in strong linkage disequilibrium (*r*^*2*^ = 0.202), we separately analyzed the effects of these genotypes on the biochemical parameters.

The presence of the CC genotype of the *APOA5* -1131T>C polymorphism was associated with a higher risk of hypertriglyceridemia [OR 3.322 (95% CI 2.421–4.559); *p*<0.001] after adjusting for age, sex, BMI, smoking, and drinking. Similarly, the presence of the T allele of the *APOA5* c.553G>T polymorphism was associated with a higher risk of hypertriglyceridemia [OR 1.956 (95% CI 1.522–2.515); *p*<0.001] after adjusting for age, sex, BMI, smoking, and drinking.

### Effects of the *APOA5* -1131T>C and *APOA5* c.553G>T polymorphisms on apoA5 levels and serum lipid profiles

No significant genotype-related differences in age and BMI were observed between controls and hypertriglyceridemic patients with the -1131T>C or c.553G>T polymorphisms (data not shown). Regarding the *APOA5* -1131T>C polymorphism, a significant association was observed between apoA5 concentrations and the -1131T>C polymorphism in controls (*p* = 0.043), with CC controls having lower values than TT controls (Table 2). Similarly, a significant association between apoA5 concentrations and the -1131T>C polymorphism was observed in hypertriglyceridemic patients (*p* = 0.027), with CC patients having lower values than TT patients. A significant association was observed between serum TG levels and the -1131T>C polymorphism. Additionally, an association of the *APOA5* -1131T>C polymorphism with lower HDL cholesterol levels was observed in controls (*p* = 0.011) and hypertriglyceridemic patients (*p<*0.001). However, significant genotype-related differences in the LDL and total cholesterol levels were not observed (Table 2).

**Table 2:** Associations of the *APOA5* -1131T>C polymorphism with apoA5 levels and serum lipid profiles in normotriglyceridemic controls and hypertriglyceridemic patients.

	*APOA5* -1131T>C													
	TG levels<150 mg/dL (*n* = 1,549)						*p*	TG levels≥150 mg/dL (*n* = 599)						*p*
	TT (*n* = 807)		TC (*n* = 648)		CC (*n* = 94)			TT (*n* = 214)		TC (*n* = 286)		CC (*n* = 99)		
Apolipoprotein A5 (ng/mL)^*∮*^	301.7	±10.4^*a*^	274.1	±9.48^*a*,*b*^	246.3	±25.4^*b*^	**0.043**	262.0	±16.8^*a*^	219.5	±11.4^*a*,*b*^	160.7	±11.6^*b*^	**0.027**
Triglyceride (mg/dL)^*∮*^	86.7	±1.07^*b*^	91.9	±1.24^*a*^	96.5	±2.98^*a*^	**0.001**	205.2	±3.69^*c*^	222.5	±4.82^*b*^	265.2	±11.3^*a*^	**<0.001**
HDL cholesterol (mg/dL)^*∮*^	56.4	±0.47^*a*^	55.1	±0.52^*a*,*b*^	52.7	±1.31^*b*^	**0.011**	48.1	±0.79^*a*^	45.8	±0.63^*a*^	43.2	±1.09^*b*^	**<0.001**
LDL cholesterol (mg/dL)^*∮*^	119.8	±1.10	121.7	±1.25	120.6	±3.78	0.526	121.9	±2.38	120.7	±2.05	114.6	±4.19	0.105
Total cholesterol (mg/dL)^*∮*^	193.3	±1.18	194.7	±1.40	192.6	±4.19	0.682	210.4	±2.49	208.2	±2.07	207.4	±4.22	0.594

Means ± SE.

^*∮*^Tested using logarithmic transformation.

*p*-values for each group were derived by one-way ANOVA. All letters indicate *p*<0.05, as derived using Bonferroni *post hoc* tests; the same letter indicates no significant difference, whereas different letters indicate significant differences.

Regarding the *APOA5* c.553G>T polymorphism, a significant association between serum TG levels and the c.553G>T polymorphism was observed in controls (*p* = 0.004) and hypertriglyceridemic cases (*p<*0.001) (Table 3). Additionally, an association of the *APOA5* c.553G>T polymorphism with lower HDL cholesterol levels was identified in controls (*p* = 0.013) and hypertriglyceridemic patients (*p =* 0.001). However, significant genotype-related differences in the levels of apoA5, LDL and total cholesterol were not observed (Table 3).

**Table 3:** Associations of the *APOA5* c.553G>T polymorphism with apoa5 levels and serum lipid profiles in normotriglyceridemic controls and hypertriglyceridemic patients.

	*APOA5* c.553G>T									
	TG levels<150 mg/dL (*n* = 1,549)				*p*	TG levels≥150 mg/dL (*n* = 599)				*p*
	GG (*n* = 1,335)		T allele (*n* = 214)			GG (*n* = 464)		T allele (*n* = 135)		
Apolipoprotein A5 (ng/mL)^*∮*^	285.4	±7.50	296.9	±18.4	0.490	234.9	±10.1	208.6	±18.4	0.158
Triglyceride (mg/dL)^*∮*^	88.5	±0.85	95.4	±2.10	**0.004**	216.2	±3.42	248.0	±8.73	**<0.001**
HDL cholesterol (mg/dL)^*∮*^	56.0	±0.37	53.5	±0.85	**0.013**	46.9	±0.51	43.8	±0.97	**0.001**
LDL cholesterol (mg/dL)^*∮*^	120.1	±0.86	123.9	±2.39	0.170	120.8	±1.63	118.3	±3.31	0.360
Total cholesterol (mg/dL)^*∮*^	193.6	±0.94	195.7	±2.63	0.526	209.3	±1.70	207.3	±3.18	0.518

Means ± SE.

^*∮*^Tested using logarithmic transformation.

*p*-values were derived from an independent *t*-test comparing the GG genotype and the T allele in each group.

### Effects of the *APOA5* -1131T>C and *APOA5* c.553G>T polymorphisms on LDL particle size, ox-LDL and MDA levels, and ba-PWV

**Fig 1:**
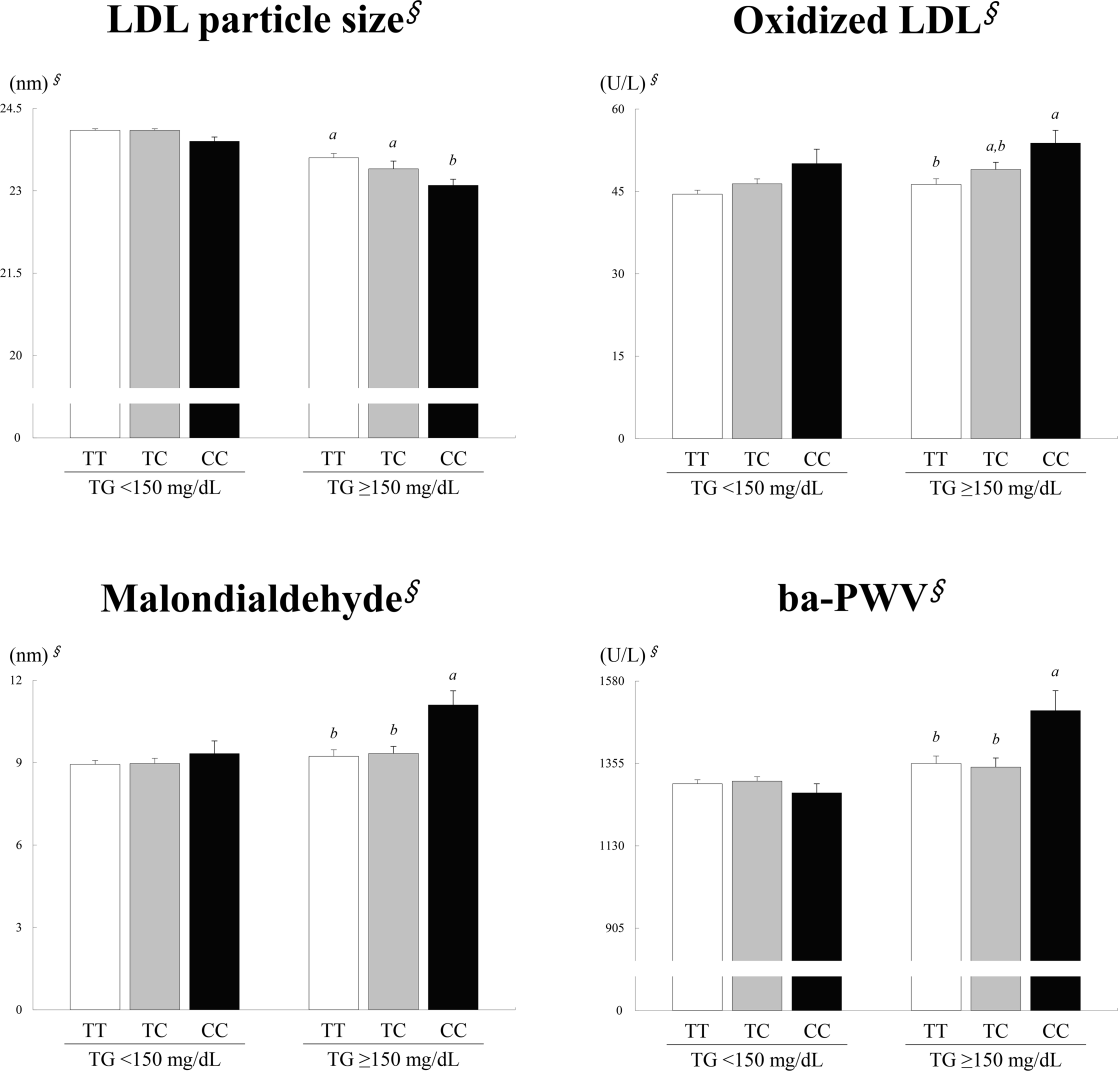
Association of the *APOA5* -1131T>C polymorphism with LDL particle size, oxidized LDL and malondialdehyde levels, and ba-PWV in normotriglyceridemic controls (TG levels<150 mg/dL) and hypertriglyceridemic patients (TG levels≥150 mg/dL). Means ± SE. ^*∮*^Tested using logarithmic transformation. *p*-values for each group were derived by one-way ANOVA. All letters represent *p*<0.05, as derived using Bonferroni *post hoc* tests after adjusting for age, sex, BMI, smoking, and drinking; differences that are not significant are marked with the same letter, and significant differences are marked with different letters.

Regarding the *APOA5* -1131T>C polymorphism, a significant association was observed between LDL particle size and the -1131T>C polymorphism in hypertriglyceridemic patients (*p* = 0.006), with patients with the CC genotype having smaller LDL particle sizes than patients with the TT or TC genotypes (Fig 1). Additionally, a significant association between ox-LDL levels and the -1131T>C polymorphism was observed in hypertriglyceridemic patients (*p* = 0.033), with patients with the CC genotype displaying higher levels than patients with the TT genotype. A significant association between MDA levels and the -1131T>C genotype was also observed in hypertriglyceridemic patients (*p<*0.001), with patients with the CC genotype displaying higher levels than patients with the TT or TC genotype. Furthermore, a significant association was observed between ba-PWV and the -1131T>C polymorphism in hypertriglyceridemic patients (*p* = 0.007), with patients with the CC genotype displaying higher levels than patients with the TT or TC genotype (Fig 1). However, for the *APOA5* c.553G>T polymorphism, no significant genotype-related differences in LDL particle size, ox-LDL and MDA levels, and ba-PWV were observed between controls and hypertriglyceridemic patients (data not shown).

### Correlation between the apoA5 concentration and lipid profiles and ba-PWV

In the total study population, apoA5 concentrations were negatively correlated with TG (*r* = -0.170, *p*<0.001) and MDA levels (*r* = -0.087, *p =* 0.001) and positively correlated with HDL cholesterol levels (*r* = 0.298, *p*<0.001) and LDL particle size (*r* = 0.193, *p*<0.001). Additionally, ba-PWV was positively correlated with TG (*r* = 0.319, *p*<0.001) and MDA levels (*r* = 0.196, *p*<0.001) and negatively correlated with LDL particle size (*r* = -0.125, *p*<0.001).

## Discussion

The -1131T>C and c.553G>T polymorphisms were associated with hypertriglyceridemia in this study population, but only the -1131T>C polymorphism directly affected apoA5 concentrations. Additionally, hypertriglyceridemic patients carrying the *APOA5* -1131T>C polymorphism displayed smaller LDL particle sizes and higher ox-LDL levels and ba-PWV, a marker of arterial stiffness [6]. Thus, the *APOA5* variants, particularly the CC genotype of the *APOA5* -1131T>C polymorphism, predispose hypertriglyceridemic patients to increased atherogenic LDL levels and arterial stiffness, probably due to the effects of the -1131T>C polymorphism on apoA5 concentrations.

The present findings of associations of the *APOA5* -1131T>C and c.553G>T polymorphisms with higher TG levels and lower HDL cholesterol levels are highly consistent with the results of meta-analyses reported by Zhao et al. [23] and He et al. [11], respectively. The apoA5 protein encoded by the *APOA5* gene is a well-known apolipoprotein and an important determinant of plasma TG levels, which are a major risk factor for CAD [3]. Two theories broadly describe the mechanism by which apoA5 modulates TG levels: (i) apoA5 inhibits the rate of very low-density lipoprotein (VLDL) production, the major carrier of TG, and (ii) apoA5 enhances the catabolism of TG-rich lipoproteins [10]. Merkel et al. [24] reported that apoA5 accelerates plasma hydrolysis of TG-rich lipoproteins by facilitating interaction with lipoprotein lipase (LPL), and apoA5 acts as an allosteric LPL activator in the lipolytic system. With respect to the c.553G>T mutation, the Gly185 amino acid residue is relatively conserved, suggesting that this residue might play a crucial role in the function of LPL. Furthermore, all c.553G>T carriers were found to have partial LPL activity, indicating that the c.553G>T mutation likely affects the LPL, thereby reducing LPL activity [25]. Huang et al. [26] investigated the effect of different amino acid substitution mutations at position 185 in apoA5 on its LPL activation properties and found that Gly at this position is important for LPL activation *in vitro*. The protein encoded by c.553G>T apoA5 consist of dimers and multimers *in vitro*, although the most of the protein is monomeric [27]. The studies with members of the LDL receptor family have indicated that c.553G>T apoA5 is functional, suggesting that defective LPL activation is the underlying mechanism [28]. Therefore, we speculate that even though the apoA5 levels were not significantly different between the GG genotype and T allele in *APOA5* c.553G>T, the T allele of apoA5 results in less LPL activation than the GG genotype.

As shown in the study by Grosskopf et al. [29], apoA5-deficient mice exhibit decreased lipoprotein lipase activity and the accumulation of larger VLDL particles, which are precursors of small dense LDLs. According to the recent study by Guardiola et al. [8], carriers of a rare allele of the *APOA5* -1131T>C polymorphism have large VLDLs and small LDLs. Hypertriglyceridemic patients with the CC genotype had significantly smaller LDL particle sizes than cases with the TT or TC genotype in the present study, despite a lack of significant differences in their LDL cholesterol concentrations. Additionally, LDL particle size was positively correlated with apoA5 concentrations.

Small dense LDLs are more susceptible to *in vitro* oxidation [30]. Moreover, changes in the LDL profile towards smaller species and decreases in serum total antioxidant levels are closely associated with increased ox-LDL levels in humans [31]. In this study, hypertriglyceridemic patients with the CC genotype of the *APOA5* -1131T>C polymorphism had smaller LDL particle sizes and higher MDA and ox-LDL levels than those with the TT genotype. MDA is an end product of lipid peroxidation. In addition to its high reactivity and toxicity, MDA is one of the most popular and reliable clinical markers of oxidative stress [32], emphasizing the relevance of this molecule to the biomedical research field. Additionally, MDA levels were positively correlated with ba-PWV, consistent with the findings reported by Kim et al. [33]. Serum MDA-LDL, which produces ox-LDL, has been reported to exert direct cytotoxicity on endothelial cells, promote the secretion of adhesion molecules, facilitate platelet aggregation and monocyte adhesion, and enhance foam cell formation in atherosclerotic lesions, all of which lead to remodeling of vessel walls and have influence on ba-PWV [34,35]. Moreover, serum levels of MDA are elevated in hypertension [36] indicating that oxidative stress is associated with BP in which has been known to be related to ba-PWV [37].

The ba-PWV has been shown to be a suitable preventative measure to screen for vascular dysfunction and the development of atherosclerosis [38]. In this study, hypertriglyceridemic patients with the CC genotype of the *APOA5* -1131T>C polymorphism had higher ba-PWV than cases with the TT or TC genotype. Additionally, LDL particle size was negatively correlated with ba-PWV. Recently, the atherogenic subclass distribution of lipoproteins has been reported to be associated with increased carotid intima-media thickness (IMT), a surrogate measure of the atherosclerotic burden [8]. Additionally, in the study by Elousa et al. [39], the -1131T>C polymorphism was significantly associated with increased common carotid IMT in obese participants in the Framingham Offspring Study.

A few limitations should be considered when interpreting the findings from the present study. Our results share the limitations of cross-sectional observational studies because we identified only associations rather than prospective predictors. Additionally, in the present study, we specifically focused on a representative group of Korean subjects. Therefore, our results cannot be generalized to other ethnic, age or geographical groups. Moreover, further studies with larger sample sizes are needed to verify the mechanism and associations of the *APOA5* c.553G>T polymorphism with apoA5 concentrations and cardiovascular disease risk factors.

Despite these limitations, *APOA5* variants, particularly the CC genotype of the *APOA5* -1131T>C polymorphism, might predispose hypertriglyceridemic patients to increased atherogenic LDL levels, smaller LDL particle sizes, higher ox-LDL levels, greater arterial stiffness, and higher ba-PWV, probably due to the effects of the -1131T>C polymorphism on apoA5 concentrations. These changes have clinical importance because hypertriglyceridemic patients with the CC genotype might exhibit subclinical atherosclerosis.
